# A Study of the Mechanical Properties of Naturally Aged Photopolymers Printed Using the PJM Technology

**DOI:** 10.3390/ma16010400

**Published:** 2023-01-01

**Authors:** Jerzy Bochnia

**Affiliations:** Faculty of Mechatronics and Mechanical Engineering, Kielce University of Technology, 25-314 Kielce, Poland; jbochnia@tu.kielce.pl

**Keywords:** 3D printing, PJM, photocurable resins, polymers, polymer aging

## Abstract

Additive manufacturing is being increasingly used both for rapid prototyping as well as the fabrication of finished components. It is important to determine how the properties of 3D printed materials change over time and how they affect the durability and usability of products. The aim of the research presented in this article was to find out what influence the natural aging period had on the mechanical properties, especially the tensile strength and modulus of elasticity, of specimens made from the selected photocurable resins using the PolyJet Matrix (PJM) technology. The tests involved determining the tensile strength and modulus of elasticity of specimens fabricated in 2013 and 2014 using two types of photosensitive resins, i.e., FullCure 720 and VeroWhite, respectively. Some of the specimens were stored under laboratory conditions until July 2022 and then tested using a universal testing machine. The experimental data obtained in 2022 for the naturally aged models were compared with those reported for the as-printed specimens. One of the main findings of this study was that the tensile strength and modulus of elasticity of the naturally aged specimens were largely dependent on the printing direction (model orientation on the build tray). The test results show that aging generally decreased the tensile strength of the specimens. In one case, however, an increase in this property was observed. For the X and Y printing directions, *R_m_* declined by 27.1% and 30.7%, respectively. For the Z direction, a decrease of only 5.5% was reported, for Full Cure 720. The modulus of elasticity of the models tested in 2022 differed considerably from that reported for the as-printed objects. Higher values of the modulus of elasticity implied that the material stiffness increased over time, and this is a common phenomenon in polymers. Interesting results were obtained for VeroWhite specimens. The modulus of elasticity decreased significantly by 25.1% and 42.4% for the specimens printed in the X and Z directions, respectively. However, for the models built in the Y direction, it increased by 27.4%. The experimental data may be of significance to users of products manufactured using the PJM method as well as to researchers dealing with the durability and reliability of such materials.

## 1. Introduction

The significant increase in the use of additive manufacturing is mainly due to rapid transformations induced by Industry 4.0. Additive manufacturing, also referred to as 3D printing, is likely to replace some machining, metal forming, casting, and injection molding operations. This is particularly true for objects with extremely complex geometries that are difficult or impossible to produce using conventional manufacturing processes. Another reason why additive manufacturing may be an alternative to some traditional operations is the fact that it is more suitable for small-batch and custom one-of-a-kind production, which is the essence of flexible manufacturing, aiming to reduce the time and cost of production as well as to keep pace with fast-changing demand patterns [1,2,3,4].

These days, additive manufacturing is becoming a desirable approach not only when specific geometries but also specific properties of solids are to be achieved. The materials used for 3D printing include liquids (e.g., photosensitive resins) and powders (e.g., polymer powders for selective laser sintering). Whichever the case, their form and properties change as a result of the 3D printing process. Thus, the solid created layer by layer on the printer build tray has characteristics different from those of the raw material. Machining, by contrast, does change the shape of the workpiece, but the material properties are practically not affected [5,6,7].

Additive manufacturing is a relatively new technology and its popularity is growing. Most studies in this area deal with the properties of 3D printed materials, e.g., [8,9,10]; 3D printed polymers are reported to be anisotropic materials with mechanical properties dependent on the object orientation on the build tray, i.e., the printing direction [11,12,13]. Studies focusing on the geometrical and dimensional accuracy of objects printed by additive manufacturing [12,14,15] reveal that the dimensions of prints may be different from the printing direction effects when compared with their CAD models [12,14,16,17]. Some investigations are devoted to the characteristics of polymer-based composite materials [17,18,19], which are commonly created by Selective Laser Sintering (SLS) or Fused Deposition Modeling (FDM)/Fused Filament Fabrication (FFF).

It is thus important to assess how the properties of additively manufactured elements change over time and, from a practical point of view, how these changes affect the durability and usability of such products. The purpose of the research described in this article was to discover how the natural aging period affected the mechanical properties, particularly the tensile strength and modulus of elasticity, of specimens made from Full Cure 720 and Vero White photocurable resins using the PolyJet Matrix (PJM) technology.

Most materials age naturally at room temperature; however, aging can also be achieved by applying special moisture or temperature conditions. Under natural conditions, polymers undergo degradation much faster than other engineering materials, e.g., metal alloys. The lower durability of polymers means that their mechanical and surface properties worsen over time. The aging processes of polymers and polymer-based composites are described, for instance, in [19,20,21,22,23,24].

Since additive manufacturing is a relatively ‘young’ technology when compared with other manufacturing processes, knowledge of the changes in the mechanical properties of 3D printed photopolymers due to physical aging is essential. The experimental results presented here can be of practical importance to users of elements built using the PJM technology as well as to researchers focusing on the durability and reliability of such products.

## 2. Materials and Methods

### 2.1. Materials

The specimens were fabricated in December 2013 and June 2014 using two different raw materials, i.e., FullCure 720 and VeroWhite, respectively. The transparent FullCure 720 resin specimens were designed in accordance with ASTM D638 [25]. For the opaque VeroWhite resin-based solids, the ISO 527 [26] standard was employed. The specimens made according to ASTM D638 were longer (165 mm) than those made in accordance with ISO 527 (75 mm). Printing long specimens in the vertical direction, i.e., printing tall and thin objects, is problematic and time-consuming. Since it was easier to follow the ISO 527 standard, shorter specimens were printed. Some of the specimens were tested immediately after printing, the rest were left to be stored under laboratory conditions (temperature 20 ± 2°C, humidity 40 ÷ 60%) for a minimum of 5 years to determine how natural aging would affect their mechanical properties. The storage period was extended to 8.5 and 8 years, respectively. In July 2022, both types of specimens were tested to measure their tensile strength. The test results were compared with the data obtained for the as-printed materials in 2013 and 2014, respectively, which are reported by previous researchers [8,9]. A total of 60 specimens were tested, with one-half representing the FullCure 720 polymer and the other half representing the VeroWhite polymer. Three different printing directions were analyzed for both materials.

### 2.2. Methods

In the PolyJet technology, model building involves jetting photosensitive liquid resin heated to 72 °C [27]. The resin, deposited layer by layer on the printer build tray, is instantly cured with UV light emitted by a lamp attached to the printer head. Some models require using support material. Prints are created along the vertical (Z) axis of the working space (i.e., the build tray also called the build platform). Layers of a polymer resin are deposited in planes parallel to the X and Y axes of the build tray. The minimum layer thickness is 16 μm [27]. The PolyJet technology uses different chemical compounds, mainly photosensitive acrylic polymer resins [28]; 3D printed models may have different properties depending on the raw material used. Generally, the raw materials are known by their trade names, e.g., FullCure 720, VeroWhite, TangoBlack, and DurusWhite. There is no classification of materials used in the PolyJet and PolyJet Matrix technologies, as is the case with metal alloys. The formulae of the raw materials and the equipment used to create objects using the PolyJet or PolyJetMatrix additive manufacturing are protected by numerous, i.e., dozens of different, patents. The PolyJet Matrix technology, also developed by Objet, is more advanced than PolyJet. It allows for the simultaneous use of two different materials to create one object with a support structure [27]. Two resins can be mixed and UV cured on the go. The resultant material is called a digital material. The name suggests a structure resembling a pixel-like pattern encountered in computer graphics. The printer head is able to simultaneously jet dozens of tiny droplets of two liquid materials to build both the model and the support structure. It is thus possible to use two different types of resins (polymers) with opposite properties, where one, for instance, ensures high stiffness (e.g., VeroWhite) and the other high flexibility (e.g., TangoBlack). The resulting mixture (the output material) will have a desirable Shore hardness, depending on the proportions of the input materials. This technology allows us to design models with different mechanical and viscoelastic properties.

The specimens analyzed in this study were printed with an Objet Connex350 system produced by Objet Geometries Ltd.—Israel using the PolyJet Matrix technology (Rehovot, Israel). Two different resins—FullCure 720 and VeroWhite produced by Objet Geometries Ltd.—Israel—were used. The printing took place in 2013 and 2014, respectively. Some of the specimens were stored under laboratory conditions until July 2022 and then tested using a universal testing machine. The experimental data obtained in 2022 for the naturally aged models were compared with those reported for the as-printed specimens.

The static tensile strength of the specimens was tested using an Inspekt mini 3kN universal testing machine produced by Hegewald and Peschke MPT GmbH—Germany equipped with LabMaster data display and evaluation software (Nossen, Germany). The tensile load was applied at a crosshead speed of 1 mm/min. The ultimate tensile strength *R_m_* was calculated by the software from the following Equation (1) [27]:

(1) $$R_{m}=\frac{F_{m}}{\bar{a}\bar{b}}$$

where: *F_m_*—maximum load, *ā*—mean measured thickness of the specimen, $\bar{b}$—mean measured width of the specimen.

### 2.3. Preparation of the Specimens

The models of the FullCure 720 and VeroWhite specimens were designed using SolidWorks CAD software in accordance with the ASTM D638 [25] and ISO 527 [26] standards, respectively. All the model data were saved as STL files to be read by the printer. Before the STL files were exported, the following settings were used: resolution—to be adjusted manually; deviation tolerance—0.016 mm, and angle tolerance—1°. The deviation and angle tolerances need to be selected carefully to make sure the curved surfaces are as accurate as possible but the file is not too big in size. A deviation tolerance of 0.016 mm is suitable because, with the Connex printer used, the horizontal build layers can be as fine as 0.016 mm. The so-called staircase effect along the radii can be reduced in this way.

**Figure 1:**
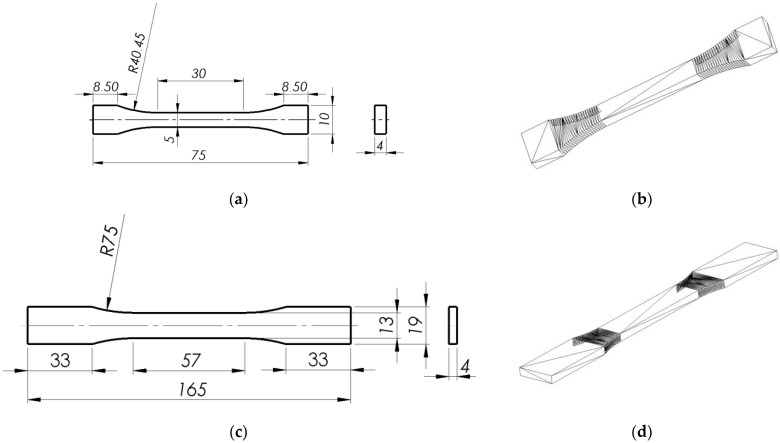
Tensile specimens, (**a**,**c**) 2D models with dimensions designed in accordance with the ISO 527 [26] and ASTM D638 [25] standards, respectively, (**b**,**d**) 3D sketches (with the polygon faces converted to triangles) saved as STL files.

Figure 1 shows 2D drawings of the two types of specimens with dimensions selected according to the relevant standards and the equivalent 3D sketches with triangulated surfaces saved in the STL format.

For identification purposes, the specimens were marked in the following way:

D—dimensions in accordance with ASTM: D638 [25],
I—dimensions in accordance with ISO 527 [26],
FC—FullCure 720 resin,
VW—VeroWhite resin,
X, Y, Z—printing directions,
1, 2, 3…—specimen numbers in a measurement series.

To illustrate how to interpret the results, the symbols should be read as follows:

– D-FC-X-4 is the fourth specimen in the series of tensile strength tests performed on FullCure 720 polymer models with dimensions according to ASTM: D638 [25] printed in the X direction,
– I-VW-Y-9 is the ninth specimen in the series of tensile strength tests performed on VeroWhite polymer models with dimensions according to ISO 527 [26] printed in the Y direction.

The FullCure 720 specimens were made in 2013, while the VeroWhite models were built in June 2014. 

## 3. Results

### 3.1. Metrology

Before the static tensile strength tests, the thickness and width of each specimen were measured at three points along the gage length using a micrometer. The two values were determined with an accuracy of up to 0.01 mm. Then, the mean thickness *ā* and the mean width $\bar{b}$ were calculated for both materials, i.e., FullCure 720 and VeroWhite polymers. These values were imported into the LabMaster universal material testing software so that the tensile strength could be calculated and the stress-strain curves could be plotted. 

The nominal thickness and the other nominal dimensions of the two types of specimen shown in Figure 1 were selected during the CAD design stage. The data were converted to STL files, which were then exported to the printer software. The differences between the nominal and actual dimensions (i.e., the dimensions of the printed specimens) were small in the order of a few hundredths of a millimeter, depending on the model orientation (printing direction). For this reason, the input data for the LabMaster software version 2.5.3.21—Germany were the mean measured width and thickness of each specimen. 

### 3.2. Tensile Tests

**Figure 2:**
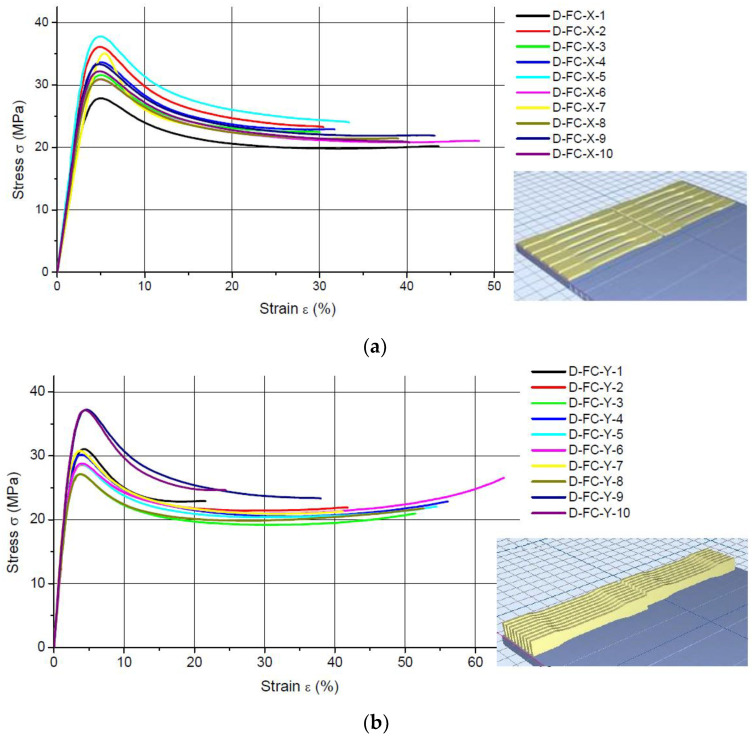

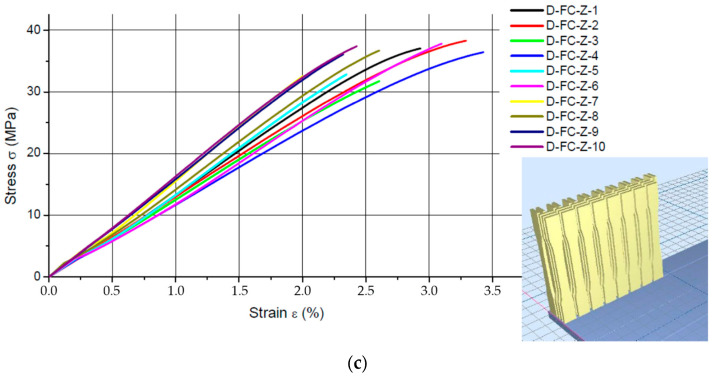
Tensile test data for naturally aged specimens printed using FullCure 720 photocurable resin in: (**a**) the X direction, (**b**) the Y direction; (**c**) the Z direction.

**Figure 3:**
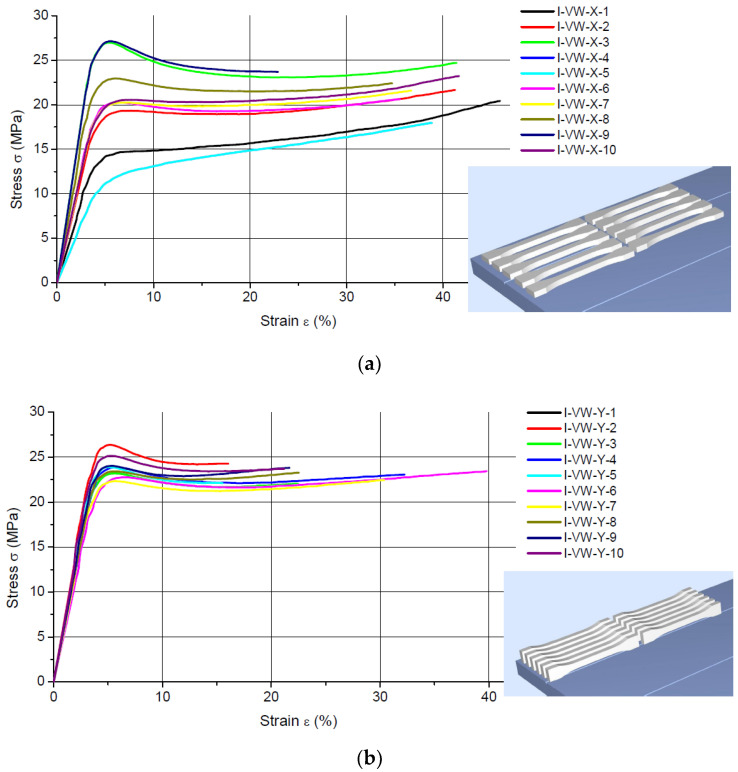

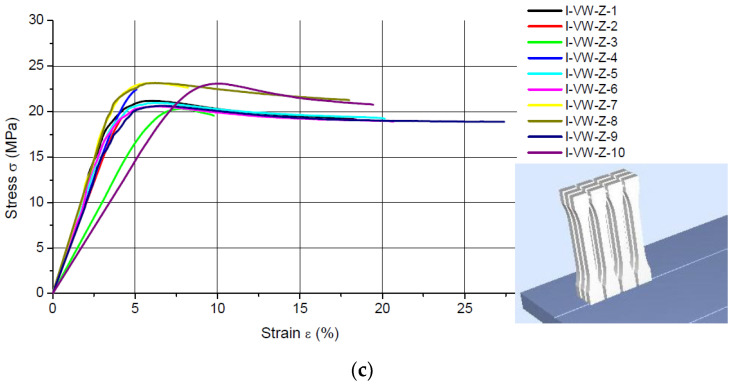
Tensile test data for naturally aged specimens 3D printed using VeroWhite photosensitive resin in: (**a**) the X direction; (**b**) the Y direction; (**c**) the Z direction.

Figure 2 and Figure 3 show the results of the static tensile tests plotted as stress-strain curves obtained for the FullCure 720 and VeroWhite specimens, respectively, built in three different printing directions.

The ultimate tensile strength and the maximum strain of the specimens made of FullCure 720 and VeroWhite resins are provided in Table 1 and Table 2, respectively. The last two rows show the mean values of the tensile strength *R_m_* and the maximum percentage strain *ε_m_* and the standard deviation for the particular measurement series.

Table 3 provides the mean values of the tensile strength in MPa reported in 2013 and 2014 for the models built in three directions X, Y and Z using FullCure 720 and VeroWhite resins, respectively. The test results, displayed mainly as stress-strain curves, are discussed in previously published articles [8,9], respectively. 

Table 4 shows the mean values of the modulus of elasticity in MPa for the as-printed and naturally aged specimens, according to the raw material used. The experimental results obtained directly after printing, i.e., in 2013 for FullCure 720 and in 2014 for VeroWhite, are compared with the data collected in July 2022 for specimens from the same series stored for 8.5 and 8 years, respectively.

It should be emphasized that the as-printed VeroWhite specimens built in the Z direction subjected to tensile tests had a linear plot and fractured at about 2.5% strain; for the respective aged specimens, the plot was also linear, but the fracture was observed at 15% strain. The tensile data obtained for the Z-axis printed FullCure 720 specimens before and after aging show that the stress-strain plot was linear; the as-printed specimens fractured at about 5% strain whereas the aged specimens fractured at 2.7% strain.

The data in Table 3 do not include results obtained in July 2022; these are given in Table 1 and Table 2.

## 4. Discussion

The main aim of the experiments was to determine the mechanical properties of 3D-printed polymers from FullCure 720 and VeroWhite resins. The tensile strength and modulus of elasticity were determined for the naturally aged specimens and compared with those reported for the as-printed specimens. The changes were analyzed to assess the durability of models made by the PJM technology. It should be emphasized that there has been little research into the influence of aging on the performance properties of materials shaped through additive manufacturing, in general, and PJM technology in particular. Most studies of the physical and mechanical properties of polymers deal with polymers made using conventional methods. The research described in this article provides information on the aging of materials obtained through a relatively new method, the PJM technology; because of its growing popularity, investigations should be continued.

**Figure 4:**
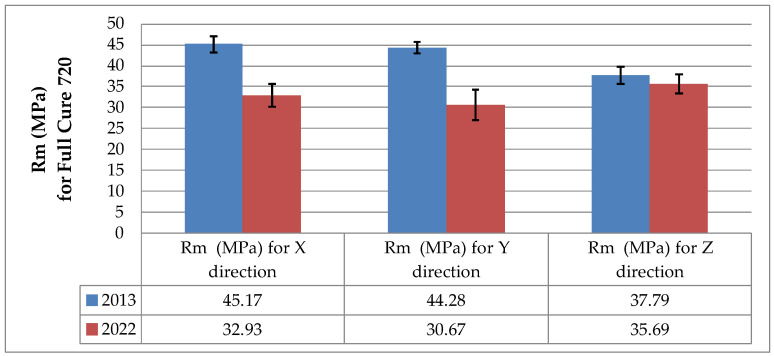
Tensile strength *R_m_* of the as-printed (December 2013) and naturally aged (July 2022) specimens based on FullCure 720 resin.

**Figure 5:**
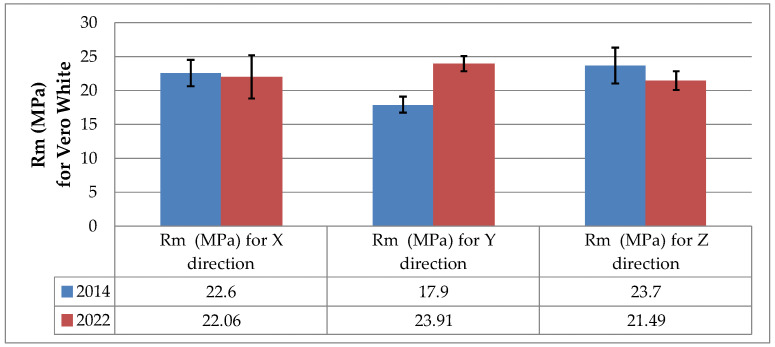
Tensile strength *R_m_* of the as-printed (June 2014) and naturally aged (July 2022) specimens based on VeroWhite resin.

Changes in the tensile strength of the 3D printed polymers resulting from aging are illustrated in the bar charts in Figure 4 and Figure 5, which were prepared on the basis of the data presented in Table 1, Table 2 and Table 3.

The results of the static tensile strength tests for the as-printed and naturally aged specimens made from FullCure 720 resin displayed in Figure 4 indicate that aging was responsible for a decrease in the ultimate tensile strength *R_m_* of this material. For the X and Y printing directions, *R_m_* declined by 27.1% and 30.7%, respectively. For the Z direction, a decrease of only 5.5% was reported.

As shown in Figure 5, the tensile strength data obtained for the VeroWhite specimens were different. An interesting observation is the fact that for models printed in the Y direction, the tensile strength increased by 25.1%. The tensile strength of the models built in the X and Z directions decreased due to aging by 2.4% and 9.3%, respectively. Similar conclusions can be drawn by comparing the stress-strain curves presented in [9] with those provided in this article.

**Figure 6:**
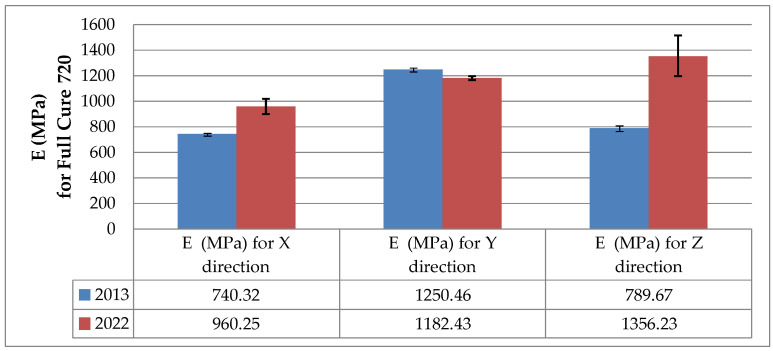
Modulus of elasticity *E* of the as-printed (December 2013) and naturally aged (July 2022) specimens based on FullCure 720 resin.

**Figure 7:**
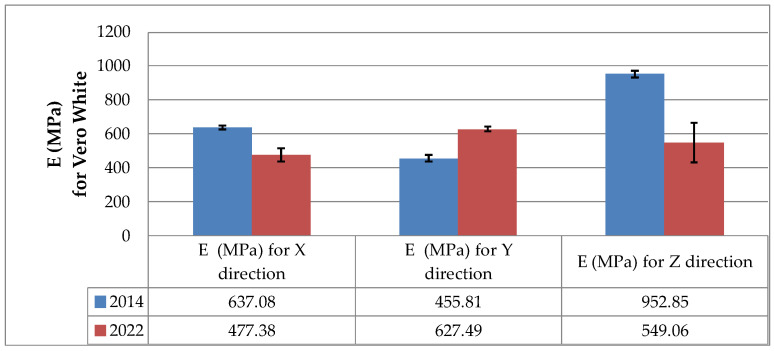
Modulus of elasticity *E* of the as-printed (June 2014) and naturally aged (July 2022) specimens based on VeroWhite resin.

Changes in the modulus of elasticity, displayed in the form of bar charts in Figure 6 and Figure 7, were calculated from the static tensile data obtained in 2014 and 2022 (Table 4).

As can be seen from Figure 6, the modulus of elasticity of the FullCure 720 models printed in the X and Z directions increased substantially after the aging process. For the specimens built in the Y direction, however, the mean modulus of elasticity dropped slightly by 5.4%. The slight decrease in the tensile strength (of only 5.5%) suggests that the specimens created in the Y orientation became less stiff. For aged polymer materials, the modulus of elasticity is predicted to rise because after some time their stiffness and brittleness increase. The experiments described in this article show how big the changes can be after several years.

The results obtained for the VeroWhite polymer (Figure 7) are different from those reported for FullCure 720. The modulus of elasticity decreased significantly by 25.1% and 42.4% for the specimens printed in the X and Z directions, respectively. However, for the models built in the Y direction, it increased by 27.4%. This rise suggests characteristic aging-induced changes, i.e., higher stiffness. The two materials are mixtures of several dozen chemical compounds [28]. The compositions are likely to have affected the aging process. Since the phenomenon was observed only for specimens printed in the Z direction, it may have been due to the occurrence of some specific adhesive bonds. Further research in this area is needed to explain this phenomenon. It should be noted, however, that the VeroWhite specimens built in the X and Z directions became more flexible after the long storage. This is visible also in the shapes of the stress-strain diagrams, especially those obtained for the Z direction (Figure 3).

This article does not analyze the maximum percentage strain because of a wide scattering of the results. The high values of the standard deviation in Table 1 and Table 2 and the shapes of the stress-strain curves in Figure 2 and Figure 3 confirm the observations.

## 5. Conclusions

1. The experiments discussed in this article aimed to determine the effects of natural aging on the mechanical properties of polymer materials additively manufactured through PolyJet Matrix technology from two types of photocurable resins, i.e., FullCure 720 and VeroWhite. The findings may be of practical importance as they help assess the durability of such materials.
2. The experimental data show that the tensile strength *R_m_* of the naturally aged specimens was dependent not only on the resin used but also on the printing direction. Aging-induced changes in the tensile strength were more visible for FullCure 720 models than for VeroWhite specimens. After the aging process, both of the 3D printed polymers were still anisotropic materials, which means their mechanical properties differed for different printing directions.
3. Lower tensile strength due to aging may suggest lower durability, i.e., a shorter service life of the 3D-printed polymer products. This drawback can be partly corrected at the design stage by selecting an appropriate orientation of the model on the build tray.
4. One of the most interesting findings of this study was that the modulus of elasticity of the specimens printed in the Z direction increased by 41.8% for FullCure 720 but decreased by 42.4% for VeroWhite.
5. From the analysis of the influence of natural aging on some of the mechanical properties of photopolymers, it is clear that further research in this field is necessary because of the increasing use of the PJM technology and PJM materials (e.g., Vero-line components used in the automotive sector).
6. The effects of aging on the behavior of additively manufactured objects need to be studied also by using climatic chambers, which offer accelerated aging conditions.

## Figures and Tables

**Table 1:** Ultimate tensile strength and the maximum percentage strain for the FullCure 720 specimens.

No.	*R_m_* (MPa)	*ɛ_m_* (%)	No.	*R_m_* (MPa)	*ɛ_m_* (%)	No.^.^	*R_m_* (MPa)	*ɛ_m_* (%)
D-FC-X-1	27.855	43.69	D-FC-Y-1	31.045	21.61	D-FC-Z-1	37.035	2.93
D-FC-X-2	36.102	30.49	D-FC-Y-2	28.607	41.86	D-FC-Z-2	38.287	3.29
D-FC-X-3	31.578	30.06	D-FC-Y-3	27.130	51.52	D-FC-Z-3	31.741	2.61
D-FC-X-4	33.634	31.76	D-FC-Y-4	30.168	56.11	D-FC-Z-4	36.427	3.43
D-FC-X-5	37.770	33.44	D-FC-Y-5	28.684	54.52	D-FC-Z-5	32.843	2.35
D-FC-X-6	30.885	48.29	D-FC-Y-6	28.794	64.08	D-FC-Z-6	37.812	3.10
D-FC-X-7	35.020	26.73	D-FC-Y-7	30.775	41.17	D-FC-Z-7	32.582	2.00
D-FC-X-8	30.939	39.10	D-FC-Y-8	27.123	52.68	D-FC-Z-8	36.701	2.61
D-FC-X-9	33.311	43.21	D-FC-Y-9	37.220	38.02	D-FC-Z-9	36.105	2.32
D-FC-X-10	32.182	40.39	D-FC-Y-10	37.115	24.51	D-FC-Z-10	37.395	2.43
$\bar{x}$	32.928	36.71	$\bar{x}$	30.666	44.61	$\bar{x}$	35.693	2.71
*SD*	2.74	6.8	*SD*	3.68	13.8	*SD*	2.38	0.5

**Table 2:** Ultimate tensile strength and the maximum percentage strain for the VeroWhite specimens.

No.	*R_m_* (MPa)	*ɛ_m_* (%)	No.	*R_m_* (MPa)	*ɛ_m_* (%)	No.^.^	*R_m_* (MPa)	*ɛ_m_* (%)
I-VW-X-1	20.429	45.91	I-VW-Y-1	23.213	22.47	I-VW-Z-1	21.186	18.42
I-VW-X-2	21.657	41.24	I-VW-Y-2	26.368	16.06	I-VW-Z-2	19.444	4.22
I-VW-X-3	26.984	41.41	I-VW-Y-3	23.213	22.47	I-VW-Z-3	20.314	9.78
I-VW-X-4	17.976	38.84	I-VW-Y-4	23.878	32.26	I-VW-Z-4	22.449	5.09
I-VW-X-5	17.976	38.84	I-VW-Y-5	23.904	15.51	I-VW-Z-5	20.953	20.17
I-VW-X-6	20.624	35.52	I-VW-Y-6	23.431	39.79	I-VW-Z-6	20.567	20.68
I-VW-X-7	21.591	36.73	I-VW-Y-7	22.463	30.40	I-VW-Z-7	23.162	8.22
I-VW-X-8	22.961	34.74	I-VW-Y-8	23.416	22.53	I-VW-Z-8	23.139	18.00
I-VW-X-9	27.133	22.89	I-VW-Y-9	24.022	21.69	I-VW-Z-9	20.639	27.40
I-VW-X-10	23.220	41.65	I-VW-Y-10	25.156	21.20	I-VW-Z-10	23.074	19.46
$\bar{x}$	22.055	37.78	$\bar{x}$	23.906	24.44	$\bar{x}$	21.493	15.14
*SD*	3.17	6.2	*SD*	1.11	7.5	*SD*	1.35	7.8

**Table 3:** Mean values of the tensile strength in MPa for FullCure 720 and VeroWhite models tested in 2013 and 2014, respectively.

Year of Test	Orientation of Specimens	*R_m_* (MPa)	Orientation of Specimens	*R_m_* (MPa)	Orientation of Specimens	*R_m_* (MPa)
2013 FullCure	X direction	45.17	Y direction	44.28	Z direction	37.79
2014 VeroWhite	X direction	22.60	Y direction	17.90	Z direction	23.70

**Table 4:** Mean values of the modulus of elasticity in MPa for as-printed and naturally aged specimens made of FullCure 720 and VeroWhite polymers.

Year of Tests and Material	Orientation of Specimens	*E* (MPa)	Orientation of Specimens	*E* (MPa)	Orientation of Specimens	*E* (MPa)
2013 FullCure	X direction	740.32	Y direction	1250.46	Z direction	789.67
2022 FullCure	X direction	960.25	Y direction	1182.43	Z direction	1356.23
2014 VeroWhite	X direction	637.08	Y direction	455.81	Z direction	952.85
2022 VeroWhite	X direction	477.38	Y direction	627.49	Z direction	549.06

## Data Availability

The data created in this study are fully depicted in the article.
